# Partial deletion of chromosome 6p causing developmental delay and mild dysmorphisms in a child: molecular and developmental investigation and literature search

**DOI:** 10.1186/s13039-021-00557-y

**Published:** 2021-07-24

**Authors:** Nikolaos Vrachnis, Ioannis Papoulidis, Dionysios Vrachnis, Elisavet Siomou, Nikolaos Antonakopoulos, Stavroula Oikonomou, Dimitrios Zygouris, Nikolaos Loukas, Zoi Iliodromiti, Efterpi Pavlidou, Loretta Thomaidis, Emmanouil Manolakos

**Affiliations:** 1 Third Department of Obstetrics and Gynecology, National and Kapodistrian University of Athens, Medical School, Attikon Hospital, Athens, GR Greece; 2 Research Centre in Obstetrics and Gynecology, HSOGE, Athens, Greece; 3 Vascular Biology, Molecular and Clinical Sciences Research Institute, St George’s University of London, London, UK; 4 Access To Genome P.C., Clinical Laboratory Genetics, Athens-Thessaloniki, Greece; 5 Department of Clinical Therapeutics, National and Kapodistrian University of Athens, Medical School, Alexandra Hospital, Athens, Greece; 6 Second Department of Pediatrics, Aglaia Kyriakou Hospital, Medical School, National & Kapodistrian University of Athens, Athens, Greece; 7 Department of Gynecology, General Hospital of Athens “G. Gennimatas”, Athens, Greece; 8 Neonatal Department, National and Kapodistrian University of Athens Medical School, Aretaieio Hospital, Athens, Greece; 9 Department of Pediatrics, School of Medicine, Aristotle University of Thessaloniki, University General Hospital AHEPA, Thessaloniki, Greece

**Keywords:** 6p22.3 deletion, Syndrome, Developmental delay, Intellectual disability, Dysmorphism, Behavioral abnormalities, High-resolution microarray analysis

## Abstract

**Background:**

The interstitial 6p22.3 deletions concern rare chromosomal events affecting numerous aspects of both physical and mental development. The syndrome is characterized by partial deletion of chromosome 6, which may arise in a number of ways.

**Case presentation:**

We report a 2.8-year old boy presenting with developmental delay and mild dysmorphisms. High-resolution oligonucleotide microarray analysis revealed with high precision a 2.5 Mb interstitial 6p deletion in the 6p22.3 region which encompasses 13 genes.

**Conclusions:**

Identification and in-depth analysis of cases presenting with mild features of the syndrome will sharpen our understanding of the genetic spectrum of the 6p22.3 deletion.

## Background

The interstitial deletion of chromosomal region 6p22.3 is a rare condition with variable phenotypic expression. To date, more than 30 children and adolescents with this deletion have been reported [1–11]. Interestingly, Colmant et al. described an electively aborted fetus with multiple abnormalities presenting the same deletion [12]. According to the size of the 6p22.3 deletion, which usually varies between 1 and 10 MB [3, 4], the clinical manifestations may include behavioral abnormalities, dysmorphic features, and structural organ defects, as well as intellectual disability.

We report herein a case of interstitial deletion of chromosome 6p investigated by array-CGH in a 2.8-year old boy with developmental delay, mild facial dysmorphism, and speech communication disorders.

## Case presentation

The patient was a 2.8-year old boy born to non-consanguineous healthy parents after an uncomplicated full-term pregnancy. He is the third child of the family, the other two offspring being a healthy 8-year old boy and a healthy 10-year old girl. He was born by cesarean section with birth weight 2.990 g (15th centile), length 50 cm (35th centile), and head circumference 34 cm (15th centile). His perinatal history was uneventful.

His motor milestones in infancy and toddlerhood are reported as normal, as he sat unsupported at the age of 6 months and walked unaided at the age of 15 months. At the age of 13 months, bilateral cryptorchidism was surgically corrected.

Due to speech and language delay, he was referred at the age of 2 years and 8 months for a full developmental assessment. On physical examination, he was found to be a sociable child with mild dysmorphic facial and body features, including frontal bossing, micrognathia, short thin nose, small deep-set eyes, small mouth with long flat philtrum, low-set ears with auricle abnormalities, widely spaced nipples, broad thumbs, and long tapering fingers. Developmental assessment showed that the child had good pretend play ability; however, verbal expression was lacking, while his comprehension was limited to simple commands. His overall developmental level was equivalent to that of a healthy 15-month old child, which corresponds to low developmental quotient (DQ = 40). On neurological examination, he showed global hypotonia of trunk and limbs without focal neurological signs. His height was 95 cm (50th centile), his weight was 15 kg (50th centile), and his head circumference was 51 cm (30th centile). Laboratory investigation, including audiological, visual, biochemical, metabolic, endocrine (thyroid, growth hormone, luteinizing hormone, follicle-stimulating hormone, adrenocorticotropin hormone and prolactin), bone age, and kidney/liver and triplex ultrasound assessments were normal. Brain MRI (magnetic resonance imaging) showed bilateral choroid plexus cysts, with a bigger cyst on the left and areas of increased signal intensity in periventricular white matter along the lateral horns of both lateral ventricles and along the frontal horns, this probably related to late myelination.

**Fig. 1:**
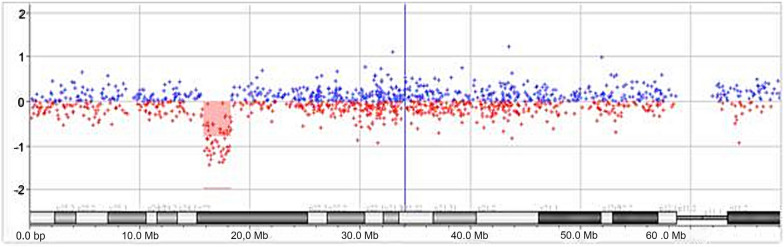
Array-CGH analysis illustrating in depth the de novo interstitial microdeletion of approximately 2.5 Mb in size on the short arm of chromosome 6 at chromosomal band 6p22.3, chr6: 15,794,379 to 18,277,334, using build GRCh37 (hg19)

High-resolution molecular karyotyping was performed with an aCGH platform of 60,000 oligonucleotides (Agilent Technologies, Santa Clara, Cal., USA) at the age of 2 years and 8 months [ISCN formula: arr[GRCh37] 6p22.3 (15,794,379_18,277,334)x1dn]. DNA extracted from blood lymphocytes showed that the 6p deletion was a 2.5 Mb deletion of the distal short arm of chromosome 6 with the proximal breakpoints between 15,794,379 bp (last deleted oligo) and 15,793,879 bp (first normal oligo), and the distal breakpoints between 18,291,461 bp (first normal oligo), and 18,277,334 bp (last deleted oligo) (Fig. 1).

Chromosome analysis with banding patterns, using GTG-banding techniques, was also carried out on stimulated blood lymphocytes and analyzed at 550–600 band resolution. Cytogenetics revealed a normal karyotype. Again, the parental karyotypes (blood lymphocytes) were normal, as produced by using banding patterns.

## Discussion

Deletions of 6p chromosome are a group of heterogeneous chromosomal anomalies. There is variability in the size and location of the breakpoints, resulting in diverse and overlapping clinical phenotypes, depending on the genes located in the deleted areas. Consequently, making an accurate genotype–phenotype correlation is often challenging. 6p deletions can involve either the distal or the interstitial part of the short arm of chromosome 6.

Deletions involving the distal part of the short arm of chromosome 6 are relatively rare. Terminal deletions of 6p24-pter have been associated with developmental delay, brain malformations (including Dandy-Walker malformation), anterior eye chamber abnormalities, hearing loss, ear abnormalities, micrognathia, and heart defects. Patients with larger sized deletions of 6p23-pter also present with microcephaly, genital anomalies, language impairment, and delayed motor development [11–18].

Interstitial deletions of 6p22-p24 have been reported even less often. Our patient had an interstitial deletion of about 2.5 Mb in chromosomal band 6p22.3. Some of the aforementioned reports concern deletions with different breakpoints which do not overlap with our case, thus exhibiting a different phenotype. For example, there is a report of four patients with a 6p22.3 deletion located more proximally in comparison with our case. Three of them had mesomelic dysplasia and the fourth (who had a larger deletion) had developmental delay without skeletal anomalies [19]. Ladinsky et al. also reported a patient with skeletal abnormalities (lower extremity hemimelia with mesomelic shortening), facial dysmorphisms, sensorineural hearing loss, and cardiac and renal abnormalities. This patient had a 6p22.3 deletion located from positions 20, 019, 758 to 21, 784, 966 [20].

Our patient presented with developmental delay and mild dysmorphisms. This phenotype is consistent with the clinical spectrum of other 6p22.3 overlapping deletions reported in the literature and comprises psychomotor delay, intellectual disability, behavioral abnormalities, dysmorphic facial features, defects in brain, heart, kidney and eye development, short neck, clinodactyly, and syndactyly.

The deleted region in our patient encompasses 13 genes, namely, *ATXN1* (ataxin-1 gene), *CAP2* (cyclise-associated actin cytoskeleton regulatory protein 2 gene), *DEK* (DEK proto-oncogene), *FAM8A1* (family with sequence similarity 8, member A1), *GMPR* (guanosine monophosphate reductase gene), *KDM1B* (lysine demethylase 1B), *KIF13A* (kinesin family member 13A gene), *MYLIP* (myosin regulatory light chain interacting protein gene), *NHLRC1* (NHL repeat containing E3 ubiquitin protein lipase 1 gene), *NUP153* (nucleoporin 153 gene), *RMB24* (RNA binding motif protein 24 gene), *STMND1* (stathmin domain containing 1 gene), and *TPMT* (thiopurine S-methyltransferase gene) (Table 1).

**Table 1:** Clinical information for all cases carrying a 6p22.3 deletion overlapping with our case

Pt	[1]	[2]	[3]a	[3]b	[3]c	[3]e	[3]f	[4]a	[4]b	[4]c	[4]d	[4]e	[5]a	[6]	[7]	[8]	[9]	[10]3	[10]4	Pr.case
Age	4	18	15	4	0	7	3	15	4	6.5	6.5	17	15	15	11	0	3.6	2.5	5	7.6
Sex	F	M	M	M	F	F	M	F	M	F	M	F	M	F	F	M	M	M	M	M
Head	−	+	−	−	−	−	−	−	−	−	−	−	−	+	−	−	+	+	−	−
Craniofacial dysmorphies	+	+	+	−	+	+	+	+	+	+	+	−	+	+	+	+	+	+	+	+
Neck/chest	+	−	+	−	+	−	+	−	−	−	−	−	−	+	−	+	+	+	+	+
Heart defect	+	−	−	na	+	−	+	−	+	−	−	−	−	+	−	−	+	−	−	+
Respiratory	−	−	−	−	+	−	−	−	−	−	−	−	−	−	+		+	−	−	−
Abdomen	−	+	−	−	+	−	−	−	−	+	−	−	+	−	−		+	−	+	−
GI	−	−	−	−	+	−	−	−	−	−	−	−	−	−	−		−	−	−	−
Kidney	−	−	−	−	−	−	−	−	−	−	−	−	−	+	+	+	−	−	−	−
Genital	−	+	−	−	−	−	−	−	−	−	−	−	−	−	−		−	+	−	−
Skeletal	−	+	+	−	+	−	+	+	+	+	+	−	+	+	−	+	+	+	+	+
Skin	−	−	+	−	+	−	−	−	−	−	−	−	+	+	−		−	−	−	−
CNS	−	+	na	na	na	−	+	+	−	−	−	−	na	−	−	+	+	+	−	−
Hypotonia	−	+	−	−	+	−	+	−	−	+	+	−	+	−	−		+	−	−	+
DD/ID	+	+	+	+		+	+	+	+	+	+	+	+	+	+		+	+	+	+
ASD	−	+	+	+		−	−	−	−	+	−	−	−	−	−		−	−	−	−
ADHD	−	−	−	−		−	−	−	−	+	−	−	−	−	−		−	−	−	−
Learning disorder	na	−	−	−		na	+	+	na	+	na	+	+	na	na		na	na	na	+

**Fig. 2:**
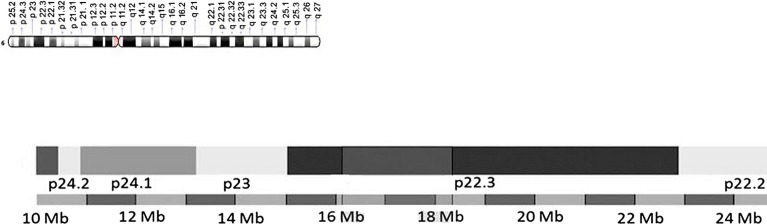
Overlapping deletions cases. For cases [3]b, [3]c, [3]e, [3]f, [5]a, [7], [8], [9], [10]3, [10]4 (see Table 2) there were no available breaking points

**Fig. 3:**
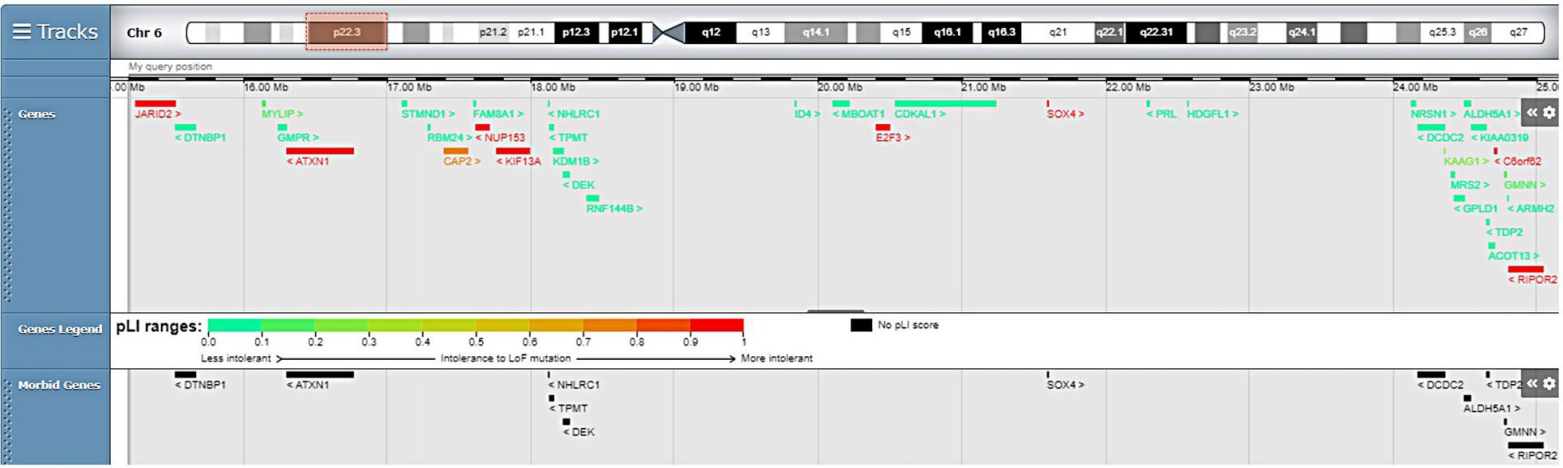
Genes and gene predictions located in the deleted region are taken from https://decipher.sanger.ac.uk. The red box indicates the deleted region of the 6p22.3 chromosome band

In the literature, there are several reports of 6p22.3 deletions which overlap with the deleted region of our patient. Our search on the PUBMED database revealed 19 cases, while the DECIPHER database revealed 18 cases. Some of these patients were genetically investigated by using FISH analysis, without accurate mapping of the breakpoint borders, as the latter technique was not available at the time of their investigation. In the interim, advanced molecular technology, such as the use of array-CGH, has allowed more precise evaluations of the breakpoint borders and identification of the included genes. During the past decade, researchers have identified critical overlapping regions in the deleted areas which could be candidates for the 6p22.3 deletion phenotype (Fig. 2 and Table 2). Bremer et al. proposed a 2.2 Mb minimal critical region involving 12 genes, while DiBenedetto et al. identified a 6p deletion of about 1 Mb, encompassing 5 genes [1, 2]. Genome.ucsc.edu indicates that the most consistently implicated genes *are MYLIP, GMPR, ATX1, NUP153, KIF13A, NHLRC1, TPMT, DEK*, and *JARID2* (Fig. 3). However, the deleted area of our patient did not include the latter gene. Apart from the number and type of genes involved in the deleted areas, other molecular mechanisms besides gene-dosage effects need to be considered in order to interpret the clinical phenotype. Such mechanisms could be the presence of modifiers in the non-deleted alleles, regulatory regions, or other genes elsewhere in the genome, as well as different penetrance or variable expressivity of HI of the deleted genes. Furthermore, the pLI score of the deleted genes, which shows intolerance to loss-of-function mutations thus indicating that haploinsufficiency of a specific gene could be responsible for clinical manifestations, should be taken into consideration. Finally, structural and quantitative chromosomal rearrangements, collectively referred to as structural variation (SV), contribute, to a large extent, to the genetic diversity of the human genome and thus are of high relevance for rare diseases, as well as for cancer and for evolutionary genetics. Recent studies have shown that SVs may not only affect gene dosage but also modulate basic mechanisms of gene regulation. SVs can alter the copy number of regulatory elements or modify the 3D genome by disrupting higher-order chromatin organization, such as topologically associated domains. As a result of these position effects, SVs can influence the expression of genes distant from the SV breakpoints, thereby causing disease. The impact of SVs on the 3D genome and on gene expression regulation must be considered when interpreting the pathogenic potential of these variant types [21].

**Table 2:** Cases carrying a 6p22.3 deletion overlapping with our case, sizes of the deletions and technique used

Case	Deletion	Size of deletion	Technique used
Present case	6p22.3	2.5 Mb	ArrayCGH
[1]	6p22.3	7.1 Mb	ArrayCGH
[2]	6p22.3	1 Mb	ArrayCGH
[3]a	6p22.3-p23	5.4 Mb	ArrayCGH
[3]b	6p22.3	1 Mb	ArrayCGH
[3]c	6p22.3-p24.3	14.6 Mb	ArrayCGH
[3]e	6p22.3	5.2 Mb	ArrayCGH
[3]f	6p22.3-p24.1	8.8 Mb	ArrayCGH
[4]a	6p22.3-p24.1	4.8–4.9 Mb	ArrayCGH
[4]b	6p22.3-p24.1	3.1 Mb	ArrayCGH
[4]c	6p22.3-p24.1	2.3–2.6 Mb	ArrayCGH
[4]d	6p22.3-p24.1	189-241 kb	ArrayCGH
[4]e	6p22.3-p24.1	116-163 kb	ArrayCGH
[5]a	6p22.1/p22.2–6p23 (FISH)	15 Mb	FISH
[6]	6p22.1–6p23	13.3 Mb	SNP oligonucleotide array
[7]	6p22.1-p22.3	NA	ArrayCGH
[8]	6p22.3-p24.3	15.2 Mb	ArrayCGH
[9]	(pter-p23:p21.33-qter) or		
(pter-p25.2:p22.2-qter)	na	Classic karyotype	
[10]3	6p22 ~ 24p22 ~ 24	na	FISH
[10]4	6p22p24	na	FISH

A discussion of the proteins and their functions encoded by the deleted genes of our case follows, as it will shed light on the clinical, developmental, and other findings presented (see also Table 3).

**Table 3:** Summary of the genes included in the deleted area of our case. Function and disease association of the genes, pLI and haploinsufficiency score and OMIM number are provided

Gene symbol	Function	Disease association	pLI score	Haploinsufficiency score	OMIM reference
*ATX1*	RNA and protein binding; transcriptional repressor activity	Spinocerebellar ataxia type 1	0.97	5,05	601,556
*CAP2*	Actin-binding protein	Human hepatocellular carcinoma	0.70	40,60	618,385
*DEK*	Chromatin-remodeling	Cancer biology, autoimmune diseases, cognitive function	0.06	4,88	125,264
*FAM8A1*	NA	NA	0.02	59,65	618,409
*GMPR*	Maintaining the intracellular balance of A and G nucleotides	Progressive external ophthalmoplegia	0.00	30,36	139,265
*KDM1B*	Lysine demethylase 1B	Flavin-dependent histone demethylases regulate histone lysine methylation, an epigenetic mark that regulates gene expression and chromatin function	0.00	35,23	613,081
*KIF13A*	Intracellular transport, neuronal signal transduction	NA	1.00	42,26	605,433
*MYLIP*	Protein–protein interaction,cell signaling, cholesterol metabolism, inhibition of neurite outgrowth	NA	0.18	34,06	610,082
*NHLRC1*	E3 ubiquitin ligase activity	Myoclonic epilepsy of Lafora	0.09	57,05	608,072
*NUP153*	Mediate the regulated movement of macromolecules between the nucleus and cytoplasm	NA	1.00	24,39	603,948
*RMB24*	Regulation of pre-mRNA splicing, mRNA stability and mRNA translation important for cell fate decision and differentiation	NA	0.09	41,96	617,605
*STMND1*	NA	NA	0.00	80,55	NA
*TPMT*	S-methylation of aromatic and heterocyclic sulphydryl compounds	NA	0.00	65,83	187,680

Ataxin-1 protein (encoded by the *ATX1* gene-OMIM #601,556) is widespread in the normal human brain, mainly within neuronal nuclei, although some Purkinje cells exhibit minor cytoplasmic components. It has been shown that spinocerebellar ataxia type 1, through mutations of the gene, causes expansion of an unstable CAG trinucleotide repeat aggregation of the mutant protein and, ultimately, neurotoxicity and neurodegeneration. It has also been proposed that *ATX1* functions as a regulator of gene expression [22]. There are so far few experimental data on the effects of an absent or nonfunctional ataxin-1 protein. However, Matilla et al. reported that mice lacking ataxin-1 showed spatial learning deficits as well as motor coordination impairments [23]. Therefore, hemizygosity for *ATX1* could contribute to the observed learning disabilities. A recent meta-analysis has additionally proposed that single nucleotide polymorphisms (SNPs) in *ATXN1* may be linked to the lower than average intelligence quotient observed in ADHD [24]. Based on the fact that mouse models have shown *ATXN1* to be of crucial importance for brain function (its absence giving rise to behavioral disorders), heterozygous deletions impacting *ATXN1* function could well be involved in both developmental delay and in autism spectrum disorders, whether alone or in conjunction with other gene deletions [3].

*MYLIP* (OMIM #610,082) is a novel ERM-like protein encoded by the *MYLIP* gene. It was determined that ERM-containing proteins, interacting with the cytoplasmic part of transmembrane proteins, connect these to the cytoskeleton in cell signaling. While playing an important role in the establishment of dynamic membrane structures, they also participate in regulation of cell proliferation, differentiation, and receptor signal transduction events [25]. *MYLIP*, in addition to having an amino-terminal ERM homology domain, also has a carboxyl-terminal RING finger: the latter is involved in regulating both the growth and survival of cells through ubiquitination [26]. A study of *MYLIP* expression during rat brain development showed that *MYLIP* is specifically localized to neuronal cells and is present in various brain regions, especially in the hippocampus and cortex. In humans, *MYLIP* is expressed in various tissues, including the brain. It has been observed that overexpression of *MYLIP* inhibits nerve growth factor-driven neurite outgrowth in neuronal PC12 cells through interaction with the myosin regulatory light chain [27]. *MYLIP* is also involved in cholesterol metabolism through regulation of the LDL receptor [28]. Given the presence of *MYLIP* in neuronal cells and its thus far identified functions in neurite outgrowth, it is reasonably hypothesized that the deletion of the *MYLIP* gene has profound effects on the neuronal cytoskeleton. Additionally, the abundant expression of *MYLIP* in almost all human tissues suggests further functions and targets for this protein.

The *GMPR* gene (OMIM #139,265) encodes guanosine monophosphate reductase: this is an evolutionarily conserved enzyme from humans to bacteria which catalyzes the conversion of the ribonucleotide GMP to IMP, the latter being a precursor ribonucleotide for the synthesis of purine nucleotides. *GMPR* is found to be highly expressed in the cytosol of skeletal and cardiac myocytes and also in renal cells. To the best of our knowledge, there is only one report in the literature so far identifying a disease-causing mutation of the *GMPR* gene, this being the study by Sommerville et al. who identified a novel heterozygous *GMPR* variant as the cause of progressive external ophthalmoplegia in an adult patient. It concerns a novel variant that is responsible for decreased *GMPR* protein levels in patients’ skeletal muscle, as well as in proliferating and quiescent cells. It is moreover linked to subtle changes in nucleotide homeostasis protein levels, while there is also evidence that it may be the cause of disturbed mtDNA maintenance in skeletal muscle [29]. Whether the absence of the *GMPR* gene in 6p22.3 deletion is associated with the hypotonia observed in the phenotype of this syndrome warrants further investigation.

The *NUP153* gene (OMIM #603,948) encodes nucleoporin 153, a protein of the nucleoporin family. Nucleoproteins are the components of nuclear pore complexes (NPCs): these are membrane-embedded channels which mediate nuclear transport across the nuclear envelope [30]. Apart from their role as constituents of NPC components, nucleoporins have recently emerged as potential regulators of chromatin organization and transcription. Furthermore, their ability to regulate gene activity does not seem to be associated with NPCs and is not linked to mediating cargo translocation across the NPC central channel. Jacinto et al. demonstrated that depletion of *NUP153* in mouse embryonic stem cells induces expression of developmental genes and results in early differentiation and loss of stem cell identity. The latter studies point to the possibility that nucleoporins have a direct role in controlling developmental transcription programs. They also indicate that *NUP153* may play a chromatin-associated role in maintenance of stem cell pluripotency through functioning in mammalian epigenetic gene silencing [31, 32].

The *KIF13A* gene (OMIM #605,433) encodes for the kinesin protein family member 13A, the kinesin proteins being ATP-dependent molecular motors moving along polarized microtubules in almost all cell types. They are hypothesized to have a significant role in neuronal signal transduction. It has been shown that the kinesin‐3 motor KLP‐4, the *Caenorhabditis elegans* homologue of human KIF13A and KIF13B, mediates axonal organization and cholinergic signaling and that strains with KLP‐4 deletion had defects in locomotive signaling [33]. It is also suggested that *KIF13A* is part of the protein-trafficking machinery and plays a role in the differential targeting of various proteins across the membrane of epithelial cells [34].

*NHLRC1* (OMIM #608,072) is a single-exon gene, located on chromosome 6p22.3, which encodes malin. Malin is a 395 amino acid protein that contains a RING and 6 NHL-repeat domains, thereby acting as an E3 ubiquitin ligase [35]. Malin interacts with laforin, and the complex of these two proteins plays a regulatory role in several cellular pathways, such as glycogen metabolism, proteolytic pathways, cellular stress response, mitochondrial homeostasis, and post-transcriptional gene regulation. Loss of function of malin and/or laforin affects neuronal function in various ways. Firstly, glycogen metabolism is impaired, which leads to accumulation of aberrant glycogen within the neuronal cells in the form of Lafora bodies, the hallmark of Lafora disease, a neurodegenerative disorder. It has been demonstrated in animal models that cells which lack malin or laforin are susceptible to autophagy impairment, increased endoplasmic reticulum stress, and reduced clearance of misfolded toxic proteins, the proteins being degraded through the ubiquitin proteasome system [36–38]. Apart from the neurodegenerative effects, lack of malin or laforin results in hyperexcitability of neuronal cells that manifests as seizures. Normal glycogen is necessary for the clearance of extracellular K, given that the astrocytic Na/K-ATPase uses ATP generated from glucose 6-phosphate, the latter originating from glycogen breakdown. Conversely, nonclearance of extracellular K leads to neuronal hypersynchronization and burst firing, the crucial mechanism of seizure generation and propagation. Furthermore, normal glycogen synthesis and breakdown are of extreme importance for the homeostasis of glutamate, the main excitatory neurotransmitter in the brain. There is hence an important connection between the accumulation of abnormal glycogen and epilepsy, which urgently requires further investigation [39].

*DEK* (OMIM #125,264) is a chromatin-remodeling gene that is expressed in most human tissues and is well known for its role in cancer biology and in autoimmune diseases. In vitro *DEK* depletion decreases cellular proliferation; it also induces DNA damage, which subsequently leads to apoptosis, and down-regulates canonical Wnt/βcatenin signaling, a molecular pathway crucial for learning and memory. There are few studies demonstrating a link between *DEK* deletion and deficits in cognitive function. Notably, a study by Ghisays et al. demonstrated that the *DEK* protein is abundantly expressed in healthy adult murine brains in corticolimbic structures, including the medial prefrontal cortex, amygdala, and hippocampus, which are linked to memory, learning, and neurogenesis [40]. To our knowledge, there is as yet no report in the literature associating lack of *TPMT* (OMIM #187,680), the enzyme that catalyzes S-methylation of aromatic and heterocyclic sulphydryl compounds, with the 6p22.3 deletion phenotype.

The phenotype of our case is similar to the other 6p22-deletion cases described in the literature. Most cases present with craniofacial dysmorphisms, learning disorders, and developmental and intellectual disabilities. There is sufficient evidence to conclude that deficits in the 6p22.3 chromosomal region result in major disruption of pathways responsible for proper early development, especially of the central neural system, with severe impairment of specific intellectual and cognitive functions. Craniofacial dysmorphisms usually accompany and reflect the anomalies of the central neural system. The clinical features seen in our case could be attributed to the lower levels and reduced activity of crucial proteins encoded by the missing genes. Thus, *ATX1* could be responsible for the speech delay, learning deficits, and low intelligence and developmental quotients. The *DEK* gene defect might act synergically, affecting both memory and learning capabilities. Reduced function of *GMPR* could lead to hypotonia, cardiac defects, and skeletal abnormalities. Nevertheless, it is well established that any phenotype penetrance depends on the presence of modifiers found in the non-deleted alleles, regulatory regions, or other genes in different locations of the genome apart from those specific genes of the missing region that are mainly responsible.

The boy herein presented will be followed up for any reduction in his developmental delay or, on the other hand, any deterioration and/or newly presenting clinical symptoms or signs, such as seizures, since the missing *NHLRC1* gene is associated with rapid and progressive adolescent-onset epilepsy. Accumulating data on such cases contribute to improvement in genetic counselling for rare and challenging hypo-chromosomal findings, which, today, are more frequently being detected thanks to constant advances in genetic analysis technologies. Moreover, enhanced accuracy in genotype–phenotype mapping will further aid in achieving ever better prognosis of outcome in the future.

## Conclusions

6p22.3 deletions are rare and there is variability in the phenotype due to the variable sizes and locations of the deletions. With this report, we describe the phenotype of a case with 6p22.3 deletion while presenting our review of the literature in an attempt to identify the implicated genes as well as their possible pathogenetic link with the clinical spectrum of this syndrome, as manifested in our case and other similar cases.

## Data Availability

Data sharing is not applicable to this article as no datasets were generated or analysed during the current study.

## Abbreviations

6p22.3 deletion: Deletion of p arm 22.3 locus of chromosome 6
array-CGH: Array comparative genomic hybridization
DQ: Developmental quotient
MRI: Magnetic resonance imaging
*MYLIP*: Myosin regulatory light chain interacting protein
*GMPR*: Guanosine monophosphate reductase
*ATXN1*: Ataxin-1
*NUP153*: Nucleoporin 153
*KIF13A*: Kinesin family member 13A
*NHLRC1*: NHL repeat containing E3 ubiquitin protein lipase 1
*TPMT*: Thiopurine S-methyltransferase
*KDM1B*: Lysine K-specific demethylase 1B
FISH: Fluorescence in situ hybridization
SNPs: Single nucleotide polymorphisms
ADHD: Attention-deficit/hyperactivity disorder
LDL: Low density lipoprotein
NPCs: Nuclear pore complexes
ATP: Adenosine triphosphate
DD: Developmental disability
ID: Intellectual disability
ASD: Autism spectrum disorder
